# N-Terminal Fragments of TDP-43—In Vitro Analysis and Implication in the Pathophysiology of Amyotrophic Lateral Sclerosis and Frontotemporal Lobar Degeneration

**DOI:** 10.3390/genes15091157

**Published:** 2024-09-01

**Authors:** Anna A. Chami, Léa Bedja-Iacona, Elodie Richard, Debora Lanznaster, Sylviane Marouillat, Charlotte Veyrat-Durebex, Christian R. Andres, Philippe Corcia, Hélène Blasco, Patrick Vourc’h

**Affiliations:** 1 Institut National de la Santé et de la Recherche Médicale (INSERM), Imaging Brain & Neuropsychiatry iBraiN U1253, Université de Tours, 37032 Tours, France; anna.chami@univ-tours.fr (A.A.C.); lea.bedja--iacona@etu.univ-tours.fr (L.B.-I.); elodie.richard@etu.univ-tours.fr (E.R.); d.lanznaster@arsla.org (D.L.); sylviane.marouillat@univ-tours.fr (S.M.); charlotte.veyratdurebex@univ-tours.fr (C.V.-D.); christian.andres@med.univ-tours.fr (C.R.A.); philippe.corcia@univ-tours.fr (P.C.); helene.blasco@univ-tours.fr (H.B.); 2 CHU de Tours, Service de Biochimie et Biologie Moléculaire, 37044 Tours, France; 3 CHU de Tours, Service de Neurologie, 37044 Tours, France

**Keywords:** ALS, FTDL, protein aggregation, post-translational modifications, TARDBP

## Abstract

Abnormal cytoplasmic aggregates containing the TDP-43 protein and its fragments are present in the central nervous system of the majority of patients with amyotrophic lateral sclerosis (ALS) and in patients with frontotemporal lobar degeneration (FTLD). Many studies have focused on the C-terminal cleavage products of TDP-43 (CTFs), but few have focused on the N-terminal products (NTFs), yet several works and their protein domain composition support the involvement of NTFs in pathophysiology. In the present study, we expressed six NTFs of TDP-43, normally generated in vivo by proteases or following the presence of pathogenic genetic truncating variants, in HEK-293T cells. The N-terminal domain (NTD) alone was not sufficient to produce aggregates. Fragments containing the NTD and all or part of the RRM1 domain produced nuclear aggregates without affecting cell viability. Only large fragments also containing the RRM2 domain, with or without the glycine-rich domain, produced cytoplasmic aggregates. Of these, only NTFs containing even a very short portion of the glycine-rich domain caused a reduction in cell viability. Our results provide insights into the involvement of different TDP-43 domains in the formation of nuclear or cytoplasmic aggregates and support the idea that work on the development of therapeutic molecules targeting TDP-43 must also take into account NTFs and, in particular, those containing even a small part of the glycine-rich domain.

## 1. Introduction

The TDP-43 protein (TAR DNA-binding protein 43Kd) is a key player in the pathophysiology of neurodegenerative diseases known as TDP-43 proteinopathies, including amyotrophic lateral sclerosis (ALS) and frontotemporal lobar dementia (FTLD) [1,2]. It is encoded by the *TARDBP* gene, which is mutated in 3–5% of familial ALS and around 3% of familial FTLD cases in Europe [3,4,5,6,7]. TDP-43 belongs to the heterogeneous nuclear ribonucleoprotein (hRNP) family. It is mainly localized in the nucleus, where it plays an important role in genome and transcriptome regulation through its action on the transcription of numerous genes, including its own, and on the processivity and maturation of transcripts [8,9]. Under physiological conditions, a small amount of TDP-43 protein is also present in the cytoplasm, where it is involved in RNA stability.

TDP-43 is composed of 414 amino acid residues. It consists of an N-terminal domain (NTD), two RNA recognition motifs, RRM1 and RRM2, and a C-terminal domain (CTD). The complete structure of the TDP-43 protein has not been crystallized due to the prion-like CTD, a highly disordered region. However, the structures of the NTD, RRM1, and RMM2 have been determined. Several studies have shown that for TDP-43 to be functional, it must be a homodimer, and NTD is involved in this homodimerization by self-interaction [10,11]. The two RRM domains, also known as the RNA-binding domain of TDP-43, are involved in its interaction with nucleic acids. Mutations identified in these domains in ALS patients are likely to disrupt its interaction with RNAs [12]. The CTD is involved in protein–protein interactions and solubility of TDP-43. This domain contains a glycine-rich domain, which has prion-like properties.

The discovery of the involvement of TDP-43 in the pathophysiology of ALS and FTDL came from the observation of TDP-43-positive inclusions in the central nervous system of patients [1,13,14,15]. Post-mortem studies show the presence of cytoplasmic protein aggregates rich in TDP-43 proteins in over 97% of ALS cases. Several studies indicate that the formation of aggregates contributes to motor neuron death and disease progression via a prion-like mechanism [16,17,18,19]. These aggregates contain, among others, the complete form of TDP-43 and cleavage products of TDP-43, such as C-terminal truncated forms of 25 and 35 kDa (TDP-25, TDP-35).

Enzymatic cleavage by proteases is one of the many post-translational modifications that TDP-43 undergoes from its synthesis and during its life in cells. This cleavage, which can occur at several sites in the TDP-43 protein, produces C-terminal fragments (CTFs) that have been extensively studied in the literature. These CTFs, in particular the CTF TDP-25 and TDP-35, have been shown to be toxic and involved in ALS and FTDL pathophysiology. These two fragments do not contain the Nuclear Localization Signal (NLS) localized in the N-term domain, leading to the accumulation of the cytoplasm and the promotion of protein aggregation [20,21,22]. Other CTFs are also capable of forming protein aggregates. Understanding these TDP-43 CTFs and their role in the pathophysiology of ALS and FTDL is essential not only to understand the etiology of the diseases but also to target TDP-43 in its various forms (total, fragments) in order to avoid the formation of aggregates or promote their clearance.

Enzymatic cleavage of the TDP-43 protein also produces N-terminal fragments (NTFs), which have been much less studied despite the fact that they may be involved in the pathophysiology of TDP-43 proteinopathies and, therefore, need to be targeted to obtain effective therapeutic approaches. It is known that the N-terminal part of TDP-43, involved in protein dimerization, facilitates protein aggregation processes in the context of diseases such as ALS. Here, we report the study on several of these NTFs of TDP-43 known to be present in the nervous system of patients, either as a result of enzymatic cleavage or truncating mutations.

## 2. Materials and Methods

### 2.1. Cell Culture

The HEK-293T (Human Embryonic Kidney) cell line (ATCC^®^ CRL-1573™) includes easily transfectable cells, and unexpectedly, they show relationships with neurons [23]. Cells were grown in Dulbecco’s Modified Eagle’s Medium (DMEM) supplemented with 5% fetal bovine serum and 1% non-essential amino acids (ThermoFisher scientific, Waltham, MA, USA) in an incubator at 37 °C in a 5% CO_2_ atmosphere.

### 2.2. Plasmid Constructions and Transfections

Full-length human TDP-43 (TDP-43WT) was amplified by PCR from adult human brain cDNA, then cloned into the mammalian expression vector Vivid Colors™ pcDNA™6.2/N-EmGFP-GW/TOPO™ (ThermoFisher scientific, Waltham, MA, USA). The truncations were generated by site-directed mutagenesis using Q5^®^ Site-Directed Mutagenesis Kit (NEB) with the plasmid GFP-TDP-43 WT as template. Primers used are in Table 1. Concerning the plasmid expressing the truncated form NTF1-374 (variant identified in ALS), it was generated from a plasmid previously generated in our laboratory [17]. The cells were transfected with plasmid by using Lipofectamine 2000 (ThermoFisher scientific, Waltham, MA, USA), according to the manufacturer’s protocol. Briefly, 3 × 10^5^ cells per well in 6-well plates were transfected with a plasmid/lipofectamine 2000 solution at a 0.5:1 ratio in Opti-MEM (Invitrogen, Carlsbad, CA, USA).

### 2.3. RT-PCR Analysis and Western Blot

For RT-PCR analysis, total RNA was extracted using the kit Direct-Zol RNA MiniPrep (Zymo Research, Irvine, CA, USA). The reverse transcription reaction was performed with the PrimeScript™RT reagent kit (Takara, Otsu, Japan) according to the supplier’s recommendations using 400ng RNA. The PCR step was performed with the Q5 High-Fidelity DNA Polymerase enzyme according to the supplier’s recommendations, followed by agarose gel electrophoresis. For Western blotting, cells were harvested 48 h after transfection and lysed with RIPA buffer containing protease inhibitor cocktail (Roche, Rotkreuz, Switzerland) and EDTA. Samples were centrifuged at 14,000× *g* for 15 min at 4 °C. Proteins were separated by Tris-glycine SDS polyacrylamide gel (concentrations ranging from 4 to 20%), transferred to polyvinylidene difluoride (PVDF, Merck Millipore, Burlington, VT, USA) membranes, and probed with primary antibodies: TDP-43 polyclonal antibody, N-terminal, and Rabbit polyclonal IgG (Proteintech, Rosemont, IL, USA; 10782-2-AP). Membranes were incubated with HRP-labeled anti-rabbit secondary antibodies (1:25,000, Thermo Scientific). Proteins were revealed using ECL (Pierce-ThermoFischer Scientific). Quantification was performed using Image Lab (Bio-rad, Hercules, CA, USA) and ImageJ software (v2.9.0).

### 2.4. Immunocytochemistry Analysis

Cells were fixed with 4% paraformaldehyde and 4% sucrose at room temperature for 20 min. The coverslips were mounted on slides using ProLong mounting medium with DAPI (Invitrogen). Cells were analyzed by fluorescence microscopy (DM5500 B, Leica Microsystems) or by confocal microscopy (SP8, Leica Microsystems, Wetzlar, Germany) in the platform PST ASB of the University of Tours, France. One hundred cells were analyzed per condition.

### 2.5. Soluble and Insoluble Protein Fractions Analysis

HEK-293T cells were seeded in six-well plates 48 h after transfection. Cells were harvested with 500 µL of cold PBS and centrifuged at 900× *g* for 10 min at 4 °C. Supernatants were removed, and pellets were suspended in lysis buffer containing RIPA buffer, protease inhibitor cocktail, and EDTA and incubated on ice for 15 min. Benzonase nuclease (250 IU) diluted in 1 M MgCl_2_ was added, and tubes were incubated for 15 min under rotation. Tubes were centrifuged at 5000× *g* for 5 min at 4 °C. The supernatants (soluble fractions) were transferred to new tubes. The pellets were suspended in 6 M urea before centrifugation at 17,000× *g* for 30 min at 4 °C, and the supernatants (insoluble fractions) were transferred to new tubes. For Western blots, proteins were denatured in Leammli Sample Buffer (BioRad, Hercules, CA, USA) with 10% β-mercaptoethanol heated at 95 °C for 5 min. A total of 30 µg of proteins were deposited per well for PAGE (4–20% gel; Mini-PROTEAN^®^ TGX). Electrophoresis was performed for 30 min at 180 V in TGS 1X (Tris Glycine SDS). After migration, proteins were transferred to a PVDF membrane (PolyVinyliDene Fluoride, Immun-Blot, Bio-Rad) using a TransBlot^®^ Turbo™ transfer system (Bio-Rad). The membrane was incubated with a 5% milk solution diluted in TBS-Tween20 (Tris SDS buffer and 0.1% Tween20) for 1 h under agitation. It was incubated overnight at 4 °C with a primary antibody diluted in 5% milk (polyclonal Anti-TDP43 10782-8-AP, ProteinTech, Rosemont, IL, USA; monoclonal anti-GFP 11814460001, Roche) under agitation and washed with TBS-Tween20, before incubation with a secondary antibody (anti-rabbit or anti-mouse IgG, HRP-conjugated, W401B or W4021, Promega) for 1 h. After washing with TBS-Tween20, membranes were revealed by Clarity Max™ Western ECL Blotting (Bio-Rad) and observed using a ChemiDoc™ tactile imaging system (Bio-Rad).

### 2.6. Viability Assays

Cell viability was measured by MTT assay or trypan blue analysis. Tests were performed on HEK-293T cells seeded in 12-well plates 48 h after transfection. For MTT assay, 0.5 ng/mL of 3-(4,5-dimethylthiazol-2-yl)-2,5-diphenyltetrazolium bromide (MTT) was added. After incubation at 37 °C for 30 min, the insoluble formazan product was dissolved in DMSO, and absorbance was measured at 570 nm (Microplate reader, Biorad, Hercules, CA, USA). For trypan blue analysis, 10 µL of trypan blue was added to 10 µL of cell suspension before counting (cell counting chamber slide, Invitrogen) in a Countess automated cell counter (Invitrogen).

## 3. Results

### 3.1. Cleavage Sites in the TDP-43 Protein

We first listed all the cleavage sites in TDP-43 reported in patients with ALS and/or FTLD. These sites were identified by mass spectroscopy, high-performance liquid chromatography, and SDS-PAGE. Twenty-four cleavage sites are shown in Table 2. They produce fragments ranging from 15 to 45 kDa. Several cysteine proteases are at the origin of these cleavages, namely caspases, calpain, and asparaginyl endopeptidase. Interestingly, several sites, mostly in the C-terminal domain of TDP-43, may carry a mutation in ALS patients, such as N291, which we identified in an ALS case [24].

### 3.2. Construction of NTFs for In Vitro Studies 

**Figure 1:**
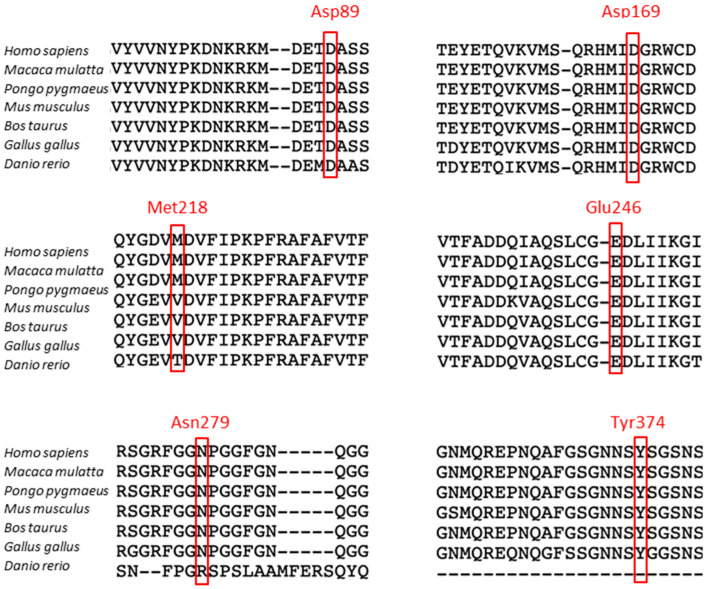
Alignments of TDP-43 protein sequences in cleavage site regions. Alignments and sequence comparisons were performed using Seaview software.

To study the functional role of TDP-43 N-terminal fragments (NTFs) generated by cleavage in vitro, we constructed vectors allowing the expression of the full-length TDP-43 protein or NTFs N-terminally fused with GFP. We selected five NTFs known to be generated in vivo by cleavage at positions 89/90, 169/170, 218/219, 246/247, and 279/280. These sites were highly conserved during the evolution of species, except in zebrafish for positions 218/219 and 279/280, using SeaView software (Figure 1) [37]. 

**Figure 2:**
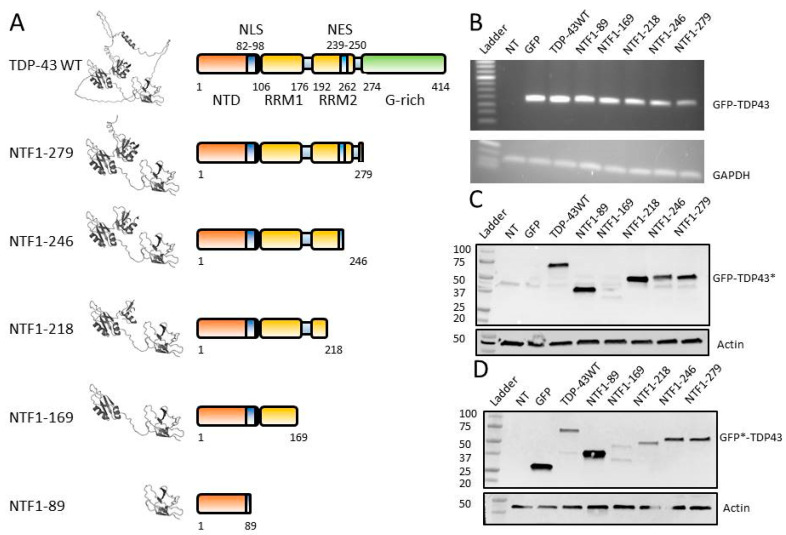
(**A**) Schematic representation (right) and 3D representation (left; obtained by AlphaFold) of the NTFs and the TDP-43WT protein. (**B**) RT-PCR analysis of expression of different constructs in HEK-293 cells after transfection (GFP and GAPDH primers for control). (**C**) Western blotting with antibody anti-N-terminal TDP-43 region (*) or Actin (control) on total proteins extracted from transfected HEK-293 cells. (**D**) Western blotting with antibody anti-GFP (*) or Actin (control) on total proteins extracted from transfected HEK-293 cells.

The five fragments were chosen on the basis of their protein domain diversity, namely absence or full-length NLS, RRM1 domain, NES, and/or RRM2 domain, as well as part of the C-terminal domain (Figure 2A). To better visualize the localization of these cleavage sites in TDP-43 and the three-dimensional structures of the NTFs produced by these cleavages, we used the 3D structure of TDP-43 (AF-Q131148-F1) available in the AlphaFold protein structure database [38,39] (Figure 2A). The N-terminal domain (NTD), which is present in all NTF constructs, comprises six β-strands and an α-helix. The NLS (positions 82–98) is located within this NTD, and it contains the 89/90 cleavage site. The NTF1-89 fragment, therefore, contains a truncated NLS. The RRM1 domain contains a β-sheet associated with two α-helices. It has a hexameric (106–111) and an octameric (145–152) nucleic acid-binding sequence. This RRM1 domain (positions 106–176) is truncated in fragment NTF1-169. The region between the two tandem RRM domains allows, due to its flexibility, these two RRM domains to be positioned in a variety of ways to bind various nucleic acids [40]. The RRM2 domain has a structure close to the RRM1 domain, with hexameric (191–198) and octameric (227–234) sequences. The NTF1-218 fragment does not contain this octameric RRM2 sequence, and the NTF1-246 fragment also shows a similarly truncated RRM2 domain. Finally, fragment NTF1-279 contains the NTD and RRM domains, as well as a few residues of the C-terminal domain. 

### 3.3. Expression of NTFs in HEK-293T

RT-PCR on RNA and Western blot experiments on protein extracts from HEK-293T cells transfected with plasmids expressing the various NTFs revealed the expression of all NTFs (Figure 2B). Expected sizes were ~27 kDa for GFP alone, ~72 kDa for GFP-TDP-43WT protein, ~36.8 kDa for NTF GFP-NTF1-89, ~45.6 kDa for GFP-NTF1-169, ~51 kDa for GFP-NTF1-218, ~54 kDa for GFP-NTF1-246, and ~55 kDa for GFP-NTF1-279 (Figure 2C,D). The GFP-NTF1-169 fragment was only visible with the anti-GFP antibody, probably due to the fact that the anti-TDP43 antibody used did not recognize this structure. The difficulty of observing this fragment in Western blots was also reported in another study [30]. 

### 3.4. Effects of NTF Expression on Aggregate Formation

**Figure 3:**
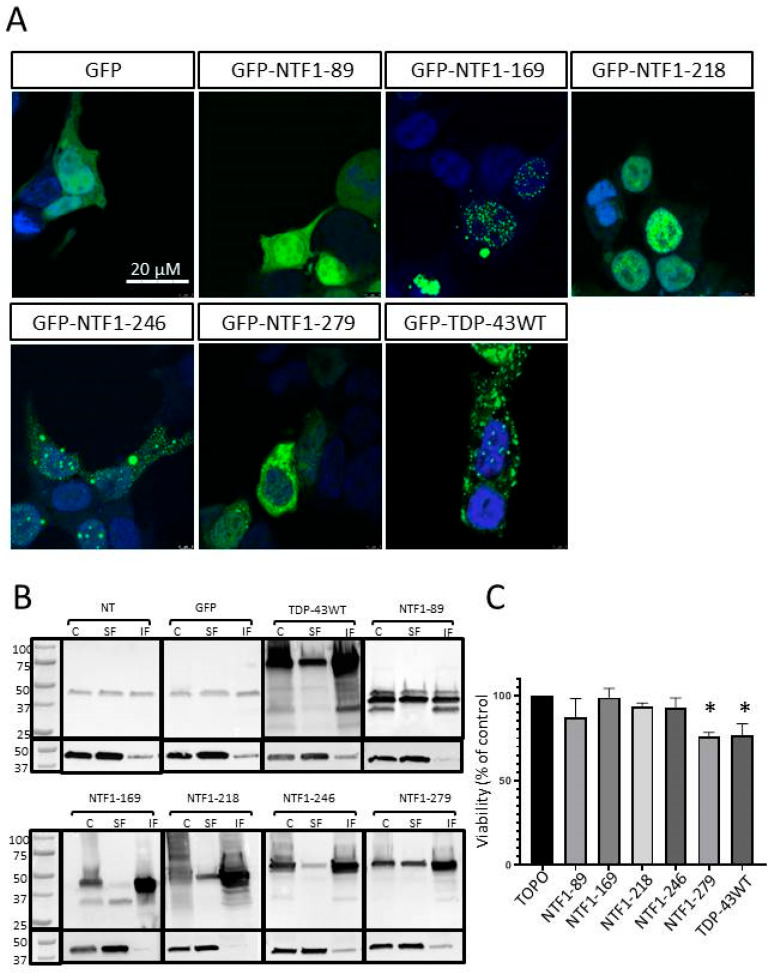
(**A**) Immunocytochemical analysis of the expression of GFP alone, NTFs, and TDP-43 WT protein fused to GFP 48 h post-transfection of HEK-293T cells. Confocal microscopy; scale bar, 20 µm. (**B**) Western blot on total protein extracts (**C**, Crude), soluble fractions (SF), insoluble fractions (IF) of non-transfected (NT) HEK-293T cells, and cells transfected by plasmids expressing GFP alone, NTFs of TDP-43, and TDP-43 WT proteins fused to GFP. Antibody directed against the N-terminal region of TDP-43. Actin was used for internal control. (**C**) Cell viability analysis of cells transfected with an empty vector (TOPO) or with plasmids expressing GFP or TDP-43 NTFs or WT protein (n = 3, * *p* < 0.05, One-Way, Dunnett’s test).

HEK-293 cells expressing GFP alone, GFP-TDP-43WT, or the various GFP-NTFs were observed by confocal microscopy 48 h post-transfection to study whether their expression led to the formation of protein aggregates (Figure 3A). Expression of GFP alone did not result in the formation of GFP-positive aggregates but rather in diffuse expressions of the protein. Expression of GFP-TDP-43WT was associated with the formation of predominantly cytoplasmic aggregates, which was in line with known data. Expression of all NTFs resulted in the formation of protein aggregates, except for the smallest NTF, the GFP-NTF1-89 fragment, which was associated with diffuse nuclear and cytoplasmic protein localization. Expression of the GFP-NTF1-169 and GFP-NTF1-218 fragments led to the formation of predominantly nuclear aggregates. GFP-NTF169 produced aggregates that could be described as puncta, while the larger GFP-NTF1-218 produced more voluminous aggregates. It should be noted that both fragments contain an NLS and no NES. The GFP-NTF1-246 and GFP-NTF1-279 fragments produced both cytoplasmic and nuclear aggregates. The GFP-NTF-246 fragment contained an NLS and a truncated NES. The GFP-NTF-279 fragment containing a complete NLS and NES, however, showed predominantly cytoplasmic aggregates, as did the wild-type form of TDP-43. 

We completed this study with an analysis of the soluble and insoluble protein fractions obtained from transfected HEK-293 cells (Figure 3B). A fraction of the endogenous TDP-43 protein expressed by non-transfected cells (NT) or cells expressing only GFP (GFP) was present in the insoluble fraction. This may be explained by the formation of transient insoluble forms in which TDP-43 is found physiologically. The insoluble fraction of cells expressing the shorter NTF GFP-NTF1-89 was not enriched in this protein. Conversely, expression of the full-length protein GFP-TDP-43WT and all other NTFs shows an enrichment of the various forms of TDP-43 in the insoluble fraction compared with the soluble fraction. Taken together, these results confirmed the observations made by microscopy, namely the presence of insoluble protein aggregates in HEK-293 cells expressing GFP-TDP-43WT and NTFs 1-169, 1-218, 1-246, and 1-279.

### 3.5. Effects of NTFs on Cell Viability

We then studied the effect of the different NTFs on cell viability using an MTT assay (Figure 3C). For this study, we removed the GFP part on protein constructs. For this purpose, another series of plasmids was generated. The MTT assay first confirmed a decrease in cell viability when cells expressed TDP-43WT protein, which is in agreement with the literature (Figure 3C). Interestingly, we observed that only the expression of the largest fragment, NTF1-279, resulted in a significant decrease in cell viability (*p* < 0.05). 

### 3.6. N-Terminal Fragment of TDP-43 Produced by Mutations in ALS Patients

TDP-43 protein is encoded by the *TARDBP* gene at 1p36.22. Truncating variants in the *TARDBP* gene are described in the ClinVar database in relation to ALS and/or FTDL, namely p.Tyr374Ter (Y374*), p.Asn378ter (N378*), and p.Trp385Ter (W385*). The two N378* and W385* variants are not linked to any publications in the database, and their involvement in these diseases remains to be strengthened. The Y374* variant is associated with several publications cited in ClinVar in relation to ALS. The insertion of an adenine in exon 6 of *TARDBP* (c.1121-1122InA) in one ALS patient from a French cohort resulted in a premature stop codon p.Tyr374Ter and the production of a truncated protein corresponding to an N-terminal fragment of the protein (NTF1-374) [41]. A c.1122 t>g substitution in an ALS patient from a UK study resulted in the same p.Tyr374Ter nonsense variant and, therefore, a truncated NTF1-374 protein [42]. Finally, a c.1119-1120delTT variant also produces this same truncated protein [43]. This variant co-segregates with ALS in a multigenerational ALS family. These variants were predicted to be pathogenic by the LRT pathogenicity prediction tool but were still considered variants of unknown significance (VUS).

**Figure 4:**
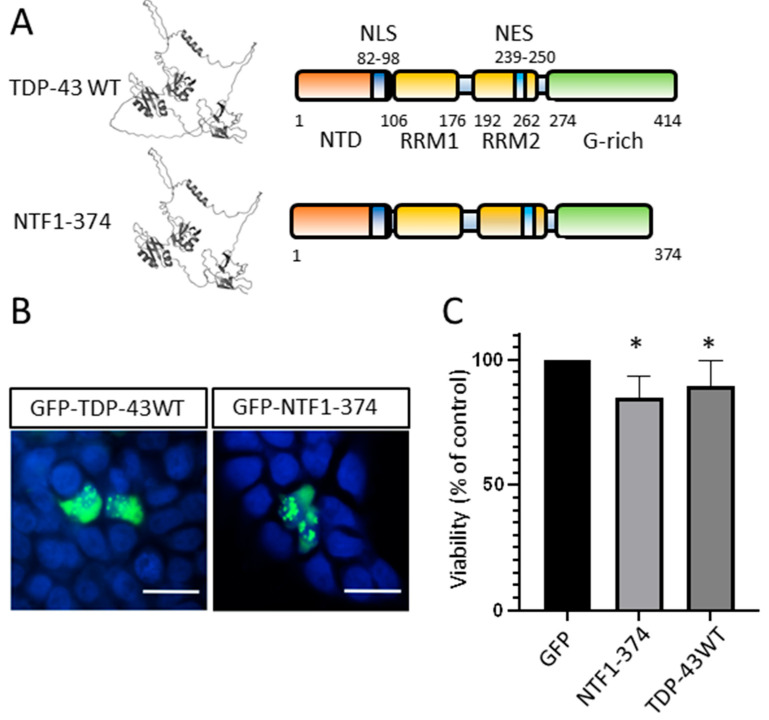
(**A**) Schematic representation (right) and 3D representation (left; AlphaFold) of the TDP-43WT protein and NTF1-374. (**B**) Immunocytochemical analysis of the expression of TDP-43 WT and NTF1-374 proteins fused to GFP, 48 h post-transfection of HEK-293 cells. Fluorescent microscopy; scale bar, 20 μm. (**C**) Analysis of viability by trypan blue assay of HEK-293 cells transfected with an empty vector or plasmids expressing GFP or TDP-43 NTF1-374 or WT proteins (n = 3, * *p* < 0.05, Kruskal–Wallis test).

A plasmid expressing this truncated GFP-NTF1-374 fragment of TDP-43 was constructed by site-directed mutagenesis (Figure 4A). We expressed this NTF in HEK-293T cells by transfection, as previously described. Then, 48h post-transfection, 69% of cells expressing the GFP-NTF1-374 fragment showed aggregates, similar to the cells expressing the GFP-TDP-43WT protein. These aggregates containing NTF1-374 or the WT form of TDP-43 showed the same cellular distribution, with a predominance of cytoplasmic localization (Figure 4B). These two cultures, expressing NTF1-374 and the TDP-43WT, also showed significantly reduced cell viability compared with the control condition expressing GFP alone (Figure 4C).

## 4. Discussion

Fragments of TDP-43 are present in protein aggregates in degenerating neurons in ALS and FTLD [18]. Here, we show that N-terminal fragments (NTFs), corresponding to TDP-43 cleavage products identified in vivo and containing NTD and at least all or part of RRM1, form protein aggregates in vitro. These aggregates appear in the cytoplasm of cells only if the NTF contains the NES region and are associated with reduced viability only if the NTF is large (at least 279 residues).

Many studies have focused on the C-terminal fragments of TDP-43 (CTFs) in ALS and FTLD, but far fewer have focused on the NTFs. This is because the C-terminal fragments are associated with major toxicity due to a pro-aggregate gain of function, and the available molecular tools prevented detection and, therefore, specific studies of the NTFs. However, there are many arguments in favor of the importance of better understanding NTFs. They appeared in molecular analyses of proteins extracted from patients’ nervous systems during post-mortem studies and carry regions involved in TDP-43 functions implicated in diseases, such as its oligomerization (N-terminal domain), dysregulation of gene expression or RNA splicing (RRM domains), and abnormal subcellular localization (NLS and NES domains) [44,45,46]. Indeed, as we proposed in a recent review, aggregation might not be the only factor involved in pathophysiology; other cellular processes might be implicated through gain or loss of function [18]. Some studies also suggest that NTFs may be pro-aggregating [30]. The present study is original because it focuses on NTFs identified in patients resulting from cleavage by proteases. All the NTFs in this study contain the NTD domain, which is of interest because the first ten residues of the NTD participate in the toxicity of the TDP-43 protein [47]. 

The shortest NTF studied, NTD1-89, contained the NTD with a truncated NLS domain. Interestingly, we showed that this fragment does not produce protein aggregates in vitro. All other NTFs form cellular aggregates but with different subcellular localization depending on the fragment. The other small N-terminal fragments, NTF1-169 and NTF1-218, mainly produce nuclear aggregates. NTF-169 aggregates are puncta-like and much smaller than NTF1-218 aggregates. Expression of the intermediate-sized fragments NTF1-246 and NTF1-279 is associated with the formation of both nuclear and cytoplasmic aggregates. A previous study showed that NTF1-243 fragments exhibited nuclear localization of aggregates in vitro [34]. The NES domain (region 239–250) is incomplete in NTF1-243 and NTF1-246. This difference in subcellular localization in the two studies could be explained by the fact that in our case (NTF1-246), the NES domain (239–250) is almost complete. The NES sequence of TDP-43 is IAQSLCGEDLII (NTF-1243, IAQLC; NTF-1246, IAQSLCGE) [48,49]. Larger NTFs showed aggregates localized predominantly in the cytoplasm. We also showed that the truncated form of TDP-43 NTF1-374 described in some ALS patients carrying heterozygous variants acts as the complete form of TDP-43 when expressed in vitro, leading to the formation of cytoplasmic aggregates. Our results provide positive evidence for a reclassification (ACMG) of the three variants producing this same truncated protein (Y374*) as probably pathogenic. Only large NTFs, i.e., NTF-279 and NTF1-374, containing at least part of the C-terminal domain, even if very small, showed a reduction in cell viability in our in vitro system. Our studies have been carried out using HEK-293 cells, which are ideal for studying sub-cellular protein localization by microscopy. It would be interesting to perform further studies, particularly on intermediate-sized NTFs, using primary neuronal cells to confirm their lack of toxicity. It would also be interesting to study these NTFs on aggregate formation and cell viability under conditions of cellular stress implicated in ALS and FTLD.

## 5. Conclusions

A better understanding of TDP-43 protein and its cleavage products is crucial, as these proteins could, via the formation of toxic cytoplasmic aggregates, be directly or indirectly involved in the pathophysiology of all cases of ALS (97% of patients with aggregates) and FTLD. Our study provides information on the N-terminal fragments of TDP-43 generated by enzymatic cleavage. We show that almost all fragments form aggregates, but only some of them have cytoplasmic localization and that the larger ones affect cell viability. Many studies in ALS and FTLD aim to identify and produce small chemical molecules or biomedicines (antibodies, intrabodies) that target TDP-43. Our results indicate that such molecules might be more effective if they also target regions of the RRM2 domain and not just the overall C-terminal region of TDP-43, which may be absent in some pathological fragments produced by cleavage in patients.

## Figures and Tables

**Table 1:** Primers used for site-directed mutagenesis.

Primers	Forward	Reverse
TDP-43WT	5′-TAGTCTGAATATATTCGGGTAACCGAAG-3′	5′-GTTCGGCACTGGATATATGAACGCT-3′
NTF1-89	5′-ATCTGTCTCATCCATTTTTC-3′	5-TAGTCATCAGCAGTGAAAGTG-3′
NTF1-169	5-TAGTCATCAGCAGTGAAAGTG-3′	5′-TATGATAGATTAGCGATGGTGTGACTGC-3′
NTF1-218	5-CCGTACTGAGAGAAGAAC-3′	5′-GGATGTGATGTAGGTCTTCATCC -3′
NTF1-246	5′-AGAGACTGCGCAATCTGA -3′	5- TTGTGGAGAGTAGTTGATCATTAAAGGAATC-3′
NTF1-279	5′-ATTACCACCAAATCTTCCAC-3′	5-TAGGGTGGCTTTGGGAATCAG-3′
NTF1-374	5′-ATAACTCTTAGAGTGGCTCTAATTCTG-3′	5′TTCCAGAACCGAAGGCCT-3′

**Table 2:** TDP-43 protein cleavage sites identified in patients with ALS and/or FTLD, with the size of the fragments generated, the enzymes identified as responsible for cleavage (if unknown, ND), and the presence of pathogenic or probably pathogenic genetic variants located at these sites.

Cleavage Sites	Fragments Size	Enzymes	References	Variants
Asp13-Glu14	42 kDa	Caspases 3, 7	[25]	No
Arg55-Leu56	43–45 kDa	ND	[26]	No
Val75-Asn76	15–20 kDa	ND	[26]	No
Asp89-Ala90	35 kDa	Caspases 3, 7	[25,27]	A90V
Val108-Leu109	30–35 kDa	ND	[26]	No
Val130-Leu131	30–35 kDa	ND	[26]	No
Asp169-Gly170	25 kDa	Caspase	[27,28]	D169G
Asp174-Cys175	25 kDa	Caspase 4	[28]	No
Cys175-Lys176	23–25 kDa	ND	[26]	No
Leu207-Arg208	<22 kDa	ND	[29,30]	No
Arg208-209	29 kDa	Caspase	[31]	No
Tyr214-Gly215	15–20 kDa	ND	[26]	No
Met218-Asp219	23 kDa	Caspases 3, 7	[30,31,32]	No
Phe229-Ala230	25 kDa	Calpain	[31,33,34]	No
Leu243-Cys244	25 kDa	Calpain	[34,35]	No
Glu246-Asp247	25 kDa	Calpain	[30,32,35]	No
Asn279-Phe280	23 kDa	Calpain	[30]	No
Gln286-Gly287	34–37 kDa	Calpain	[31,33,34]	G287S
Asn291-Ser292	35 kDa	AEP	[26,36]	N291H
Gly295-Gly296	34–37 kDa	Calpain	[31,33,34]	G295C,R,S
Ala297-Gly298	34–37 kDa	Calpain	[31,33,34]	G298S
Asn-306-Met307	32 kDa	AEP	[36]	No
Met323-Ala324	37 à 39 kDa	Calpain	[31,33,34]	No
Phe401-Gly402	ND	ND	[30]	No

## Data Availability

No new data were created or analyzed in this study. Data sharing is not applicable to this article.
